# Trends in Representation of Female Applicants and Matriculants in Canadian Residency Programs Across Specialties, 1995 to 2019

**DOI:** 10.1001/jamanetworkopen.2020.27938

**Published:** 2020-11-24

**Authors:** Gianni R. Lorello, Julie K. Silver, Genviève Moineau, Kristian McCarthy, Alana M. Flexman

**Affiliations:** 1 Department of Anesthesia and Pain Medicine, University Health Network, Toronto Western Hospital, Toronto, Ontario, Canada; 2 Department of Anesthesiology and Pain Medicine, University of Toronto, Ontario, Canada; 3 The Wilson Centre, Toronto, Ontario, Canada; 4 Women’s College Research Institute, Toronto, Ontario, Canada; 5 Department of Physical Medicine and Rehabilitation, Harvard Medical School, Boston, Massachusetts; 6 Massachusetts General Hospital, Boston; 7 Brigham and Women’s Hospital, Boston, Massachusetts; 8 Spaulding Rehabilitation Hospital, Boston, Massachusetts; 9 Emergency Medicine, Children’s Hospital of Eastern Ontario, Ottawa, Ontario, Canada; 10 Department of Pediatrics, University of Ottawa, Ontario, Canada; 11 Association of Faculties of Medicine of Canada, Ottawa, Ontario, Canada; 12 Faculty of Medicine, University of Toronto, Ontario, Canada; 13 Department of Anesthesiology, Pharmacology and Therapeutics, University of British Columbia, Vancouver, British Columbia, Canada

## Abstract

**Importance:**

Disparities in representation between sexes have been shown at multiple career stages in medicine despite increasing representation in the overall physician workforce.

**Objective:**

To assess sex representation of applicants to the Canadian R-1 entry match for postgraduate training programs from 1995 to 2019, comparing distribution between different specialties as well as applied vs matched applicants.

**Design, Setting, and Participants:**

This cross-sectional analysis of aggregate data provided by the Canadian Resident Matching Service between 1995 and 2019 analyzed aggregate data for the Canadian R-1 residency match from 1995 through 2019.

**Exposures:**

Applicant sex as reported in the Canadian Resident Matching Service database.

**Main Outcomes and Measures:**

The sex representation of applicants was compared and the longitudinal trends in sex representation were analyzed by specialty between 1995 and 2019. The sex representation of overall applicants to the Canadian R-1 entry match were compared with matched applicants, and both were stratified by specialty.

**Results:**

A total of 48 424 applicants were identified (26 407 [54.5%] female applicants), of which 41 037 were matched applicants. Using specialty groupings, female applicants were most highly represented in obstetrics and gynecology (1776 of 2090 [85.0%]) and least represented in radiology (658 of 2055 [32.0%]). Within individual subspecialties, female applicants had the lowest representation in neurosurgery (90 of 394 [22.8%]). While female applicants represented an increasing proportion of the overall applicant population between 1995 and 2019 (*z* = 2.71; *P* = .007), significant increases were seen in some, but not all, individual specialties. Differences by sex were found among Canadian medical graduate match rates to their top-ranked specialty: female applicants had a lower likelihood of being rejected for family medicine (rejection of male applicants: OR, 0.46; 95% CI, 0.39-0.54; *P* < .001) and psychiatry (OR, 0.59; 95% CI, 0.46-0.76; *P* < .001) and were more likely to be rejected for all-encompassing surgery (acceptance of male applicants: OR, 1.19; 95% CI, 1.10-1.28; *P* < .001).

**Conclusions and Relevance:**

Increasing representation of female residency applicants over time was seen in some, but not all, medical specialties in Canada, and sex-based differences in successful match rates were observed in some specialties. The reasons for these disparities require further investigation for corrective strategies to be identified.

## Key Points

**Question:**

What are the sex-based differences in Canadian residency applications and matching?

**Findings:**

This cross-sectional analysis of aggregate data from the Canadian Residency Matching Services database revealed a total of 48 424 applicants between 1995 and 2019. Female applicants had the highest representation in obstetrics and gynecology and the lowest representation in neurosurgery.

**Meaning:**

In this study, representation of residency applicants by sex varied among specialties.

## Teaser

This cross-sectional analysis examines the representation of female medical school graduates in residency programs by specialty using aggregate data from the Canadian Residency Matching Service database.

## Introduction

Sex disparities have been shown at multiple levels of female physicians’ careers, despite increasing representation in the physician workforce. A 2006 study^1^ focusing on sex demonstrated that the majority of physicians working in pediatrics, obstetrics and gynecology, and family medicine are female. In 2003, 75% of Canadian physicians between the ages of 45 to 65 were male^2^; today, more than 50% of medical school matriculants are female. Analyzing data from the Canadian Residency Matching Services (CaRMS), Baerlocher and Detsky^2^ determined that between 1995 and 2004, the odds ratio of being rejected from a first-choice residency program was 1.6-fold greater for male applicants than for female applicants. Furthermore, psychiatry, family medicine, and emergency medicine showed statistically significantly increased odds of rejection for men.^2^ Although increasing attention has been paid recently to gender disparities within the medical profession, what is lacking is a contemporary and updated analysis of applicants to the Canadian R-1 entry match for postgraduate training positions to identify whether disparities in sex representation occur at this stage of career in specialty choice and whether applicant success within the medical specialties varies by sex.

This study sought to examine sex representation in different medical specialties of matched R-1 entry match applicants from Canadian medical schools between 1995 and 2019. Secondarily, we aimed to analyze sex differences among successful match rates between different specialties.

## Methods

We conducted a retrospective analysis of the sex of Canadian medical graduate (CMG) applicants to the R-1 entry match (ie, first year of residency after matriculating from medical school) between 1995 and 2019. We obtained institutional research ethics board approval from the University Health Network Research Ethics Board with a waiver of informed consent because the study used publicly available aggregate data. This study abided by the Strengthening the Reporting of Observational Studies in Epidemiology (STROBE) reporting guideline.

### Data Sources

We extracted publically available sex-stratified aggregate data from CaRMS database from 1995 to 2019.^26^ Data were analyzed in January 2020. Prior to 1995, CaRMS did not collect applicant sex as part of their match-related data.

### Study Population

We included matched and unmatched CMG applicants to the R-1 specialty match between 1995 and 2019, inclusively. Specialty choice was defined as the applicant’s first-ranked choice. We included only CMGs because the processes for international medical graduates are distinct from those of CMGs.

### Data Collection and Outcome Measures

We extracted the following information: (1) aggregate data of both male and female applicants applying to a top-ranked chosen specialty as well as those who matched to their top-ranked specialty; and (2) sex-stratified CMGs. Only sex binaries (ie, male or female) are provided in the CaRMS database.

### Specialty Groups

Specialties were considered to be independent of each other, and specialties with less than 5 applicants per year were not presented individually to maintain anonymity. Specialties were collapsed into the following groups for ease of analysis, with specialities with less than 5 applicants matched per year excluded from individual analysis or grouped together as noted to maintain anonymity: (1) surgical specialties (cardiac surgery, general surgery, neurosurgery, ophthalmology, orthopedic surgery, otolaryngology, plastic surgery, thoracic surgery, urology, and vascular surgery) were analyzed both as an overall group and as individual specialities (thoracic and vascular surgery both had <5 matched applicants per year and trends were not presented individually); (2) pathology and laboratory medicine subspecialties were grouped together because of the low and variable numbers of applicants to individual subspecialties (ie, anatomical pathology, general pathology, laboratory medicine, medical biochemistry, medical microbiology, and neuropathology); (3) medical genetics was grouped with pediatrics because of the small absolute number of applicants to this specialty; (4) internal medicine was grouped with dermatology, as well as occupational health; (5) family medicine was grouped with community/public health; and (6) diagnostic radiology was grouped with nuclear medicine. We also note that neurology programs in Canada incorporate both adult and pediatric neurology training.

### Statistical Analysis

Specialty-stratified data were analyzed using percentages. Trends in sex representation over the study period were analyzed using a nonparametric test for trend.^3^ Logistic regression was used to analyze the relationship between sex and successful first choice. *P* < .05 was considered statistically significant in 2-tailed tests, and data analysis was performed using R version 3.6.0 (R Project for Statistical Computing) and STATA version 12.1 (StataCorp).

## Results

### Study Population Characteristics

The final study population included a total of 48 557 CMG applicants to the R-1 entry match, including 41 131 applicants who were successfully matched to their first-choice specialty. Of all CMG applicants, 26 460 (54.5%) were female, and of the CMG successfully matched applicants, 22 808 (55.5%) were female. There was an average of 1942 (range, 949-2915) applicants per year over the 25-year period analyzed.

Data were missing between 1995 and 1997, inclusively, for the following subspecialties: anatomical pathology, general pathology, hematological pathology, neuropathology, medical biochemistry, medical microbiology, medical genetics, surgical specialties, nuclear medicine, occupational medicine, community and public health, dermatology, physical medicine and rehabilitation, and radiation oncology. Several specialties reported data for limited years of the data set: medical biochemistry from 2005 to 2012; neuropathology from 1998 to 2007; occupational medicine from 1998 to 2006; thoracic surgery from 1998 to 2002; and vascular surgery from 2012 to 2019. This observation likely reflects ongoing changes to programs participating in the R-1 match process. Other random missing data points were minimal and included general pathology (5), hematological pathology (4), laboratory medicine (3), and medical microbiology (1). Finally, applicants could list their specialty category as other from 1995 through 1997; these respondents (133 applicants; 94 applicants matched) were omitted from the analysis as the specialty could not be determined, leaving 48 424 total applicants (26 407 [54.5%] female) and 41 037 matched applicants for the final analysis (eTable in Supplement).

### Sex Representation in Applicants by Specialty

**Figure 1.**
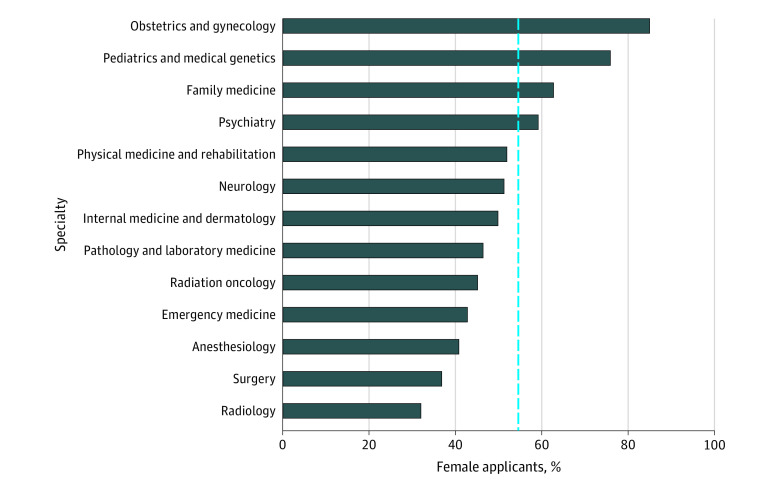
Percentage of Female Canadian Medical Graduate R-1 Entry Match Applicants to Each Specialty Group The blue reference line indicates the overall percentage of female applicants in the data set (54.5%).

**Figure 2.**
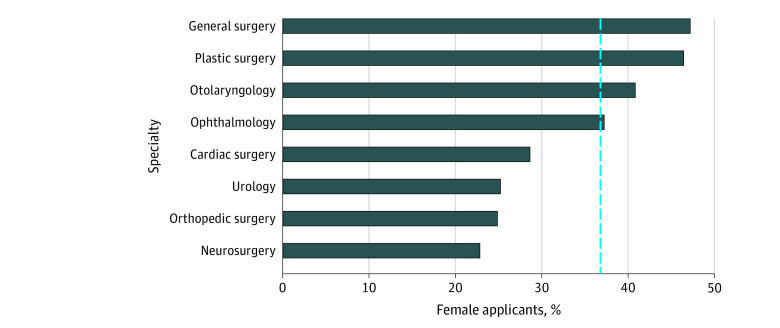
Percentage of Female Canadian Medical Graduate R-1 Entry Match Applicants Matched to Each Surgical Subspecialty The blue reference line indicates the overall percentage of female applicants matched to surgery (36.8%).

The overall proportion of female applicants varied by specialty (Figure 1; eAppendix in the Supplement). Specialties with a higher than average proportion of female applicants included: obstetrics and gynecology (1776 of 2090 [85.0%]), pediatrics and medical genetics (2427 of 3198 [75.9%]), family medicine (10 333 of 16 473 [62.7%]), and psychiatry (1663 of 2810 [59.2%]). In contrast, specialty groupings with the lowest proportion of female applicants included radiology (658 of 2055 [32.0%]), surgery (2711 of 7357 [36.8%]), anesthesiology (1005 of 2463 [40.8%]), and emergency medicine (716 of 1673 [42.8%]). There was considerable variation in the representation of female applicants to the following surgical subspecialties: general surgery (901 of 1909 [47.2%]), plastic surgery (423 of 911 [46.4%]), otolaryngology (303 of 742 [40.8%]), vascular surgery (34 of 88 [38.6%]), ophthalmology (382 of 1026 [37.2%]), cardiac surgery (59 of 206 [28.6%]), urology (195 of 773 [25.2%]), orthopedic surgery (324 of 1303 [24.9%]), and neurosurgery (90 of 394 [22.8%]) (Figure 2).

**Figure 3.**
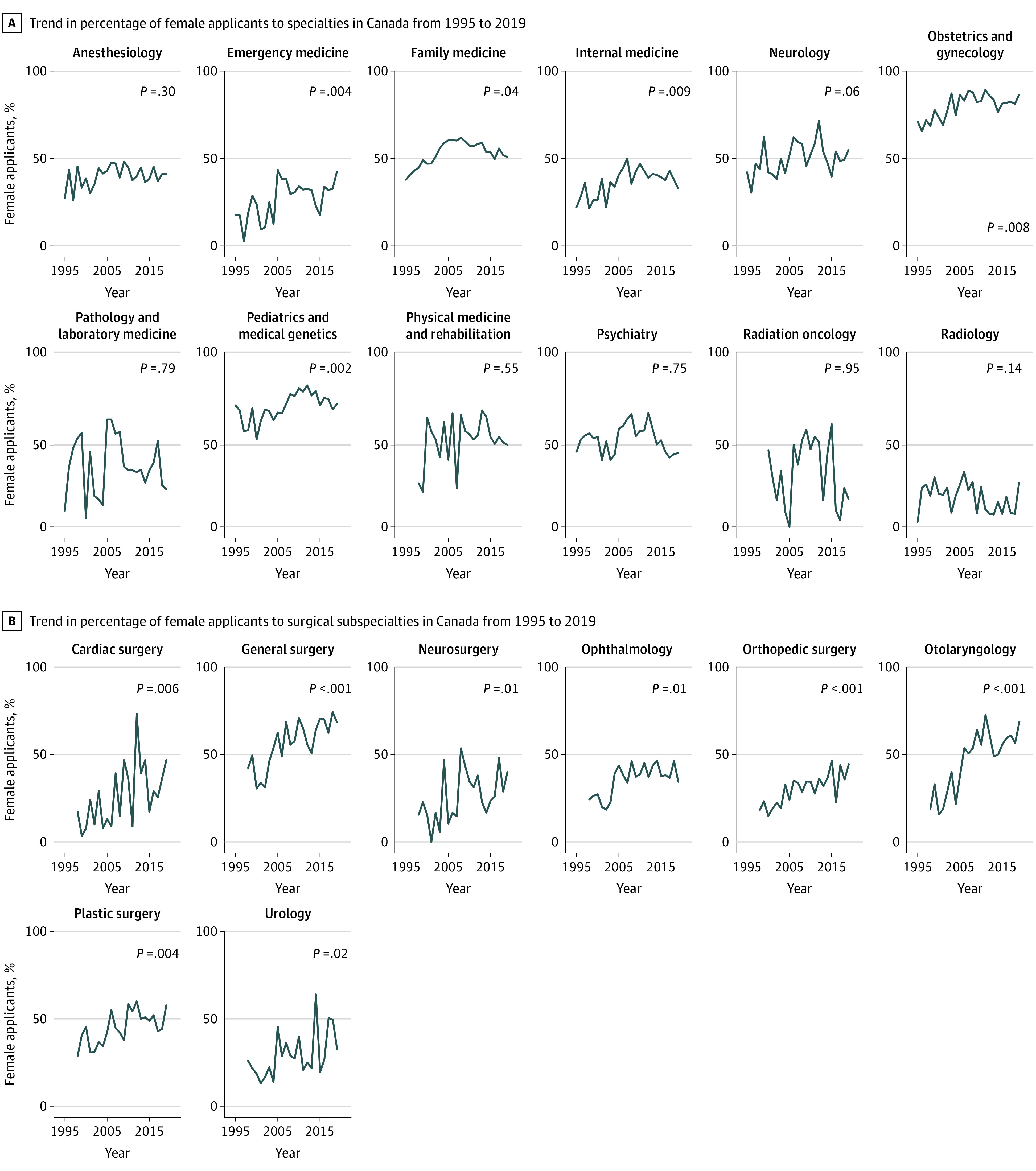
Trend in Percentage of Female Applicants to Specialties in Canada From 1995 to 2019

We observed an overall increase in female applicants to the R-1 entry match between 1995 and 2019 (*z* = 2.71; *P* = .007) as well as within several, but not all, specialties (Figure 3). Within individual surgical subspecialties, significant increases in female applicants over the study period were seen in all surgical specialties (Figure 3).

### Successful Match Rates to First-Ranked Specialties

**Figure 4.**
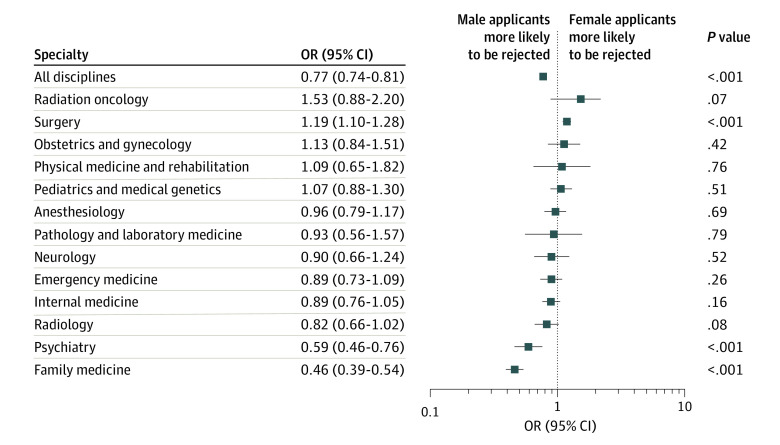
Odds Ratio of Female:Male Canadian Medical Graduate Applicants Not Matched to Their Top-Ranked Specialty From 1995 to 2019

The percentage of applicants successfully matched to their first-choice specialty, stratified by specialty, is provided in Figure 4. Overall, male applicants were more likely to be unsuccessfully matched than female applicants, although variation existed within subspecialties. Female applicants were less likely to be successfully matched to surgery (acceptance of male applicants: OR, 1.19; 95% CI, 1.10-1.28; *P* < .001) and more likely to be successfully matched to family medicine (rejection of male applicants: OR, 0.46; 95% CI, 0.39-0.54; *P* < .001) and psychiatry (OR, 0.59; 95% CI, 0.46-0.76; *P* < .001).

## Discussion

We examined sex-related differences in Canadian medical residency applicants using the most comprehensive residency matching services database available to date. We identified wide disparities in the representation of female applicants in subspecialties, with obstetrics and gynecology, pediatrics and medical genetics, and internal medicine having high rates of female applicants matched compared with specialties such as radiology, surgery, and radiation oncology. Increases in female matched applicants over time also varied considerably, with some specialties showing a consistent increase and others not. Finally, we detected a difference by sex for successful match rates to first-ranked specialty, with female applicants being more likely to match overall but less likely to match to a surgical specialty. These observations suggest that sex plays a role in specialty choice and in program selection of successful applicants. Our results do not address the reasons behind these observations, which are important next steps for research.

Although female applicants make up the majority of the R-1 entry match over the 25 years examined in our study, we found wide variation in representation by specialty as well as within surgical subspecialties. We did not study causality; however, these findings are almost certainly multifactorial and described in other reports.^4,5,6^ For example, expected work hours and flexibility varies across different specialties. Since female physicians spend a disproportionate amount of time on domestic work and parenting compared with male physicians,^7^ they may be more inclined to select specialties with more flexibility. Female applicants were also more likely to attribute increased significance to additional factors such as culture, inclusion, and diversity more than men in a previous study.^8^ An increase in the visibility of female physicians within specialties and the percentage of their representation as program directors within a specialty have been shown to be correlated with the percentage of female physicians entering a specialty.^9,10^ Moreover, medical students’ exposure to sex discrimination influences female students’ choice of specialty more than for male students; as a result, female medical graduates may be less likely to pursue an academic career.^11,12^ Evidence suggests that the surgical culture is changing,^13,14,15,16^ potentially accounting for the gradual increase in female physicians seen in all surgical subspecialties. More concerning are the differences in successful match rates among male and female applicants in surgery, psychiatry, and family medicine. Some literature^17,18,19,20,21^ suggests the fact that male applicants are more likely to match to male-dominated specialties, and female applicants to female-dominated specialties, may reflect stereotyping, culture and climate, exposure to the specialty, and/or mentoring, although these deserve more investigation.

Program directors and residency program match committees should be cognizant of inherent personal, though often unconscious, bias (ie, implicit bias) as well as structural or systemic biases when reviewing medical student applications.^22,23,24,25^ Increasing representation of all sexes and genders at a faculty level may increase medical student interests as they are able to witness role models and picture themselves in these positions. Over a 10-year period (2010-2019), 74.2% of 388 027 R-1 entry match applicants chose cultural diversity of the town or city in which they would be embarking on residency as the most influential factor in their first-choice program location.^26^

The effect of the novel coronavirus disease 2019 pandemic on workforce disparities has yet to be fully revealed, but it is predicted that progress in gender equity will suffer.^27^ For female medical graduates in medicine, many will be at a more marked disadvantage regarding pay and promotion disparities.^28^ While not known, it is plausible that virtual interviews for medical school and other positions will promote higher levels of implicit bias for female applicants and/or racial minorities.^29^

### Limitations

This study has several limitations. First, this study is limited by the nature of the data available from the CaRMS database. Although sex and gender exist on a spectrum, CaRMS reports sex in binary terms; therefore, we decided to stay consistent with the collected sex-related data and report it as such. However, this study of sex-related representation is relevant to the gendered phenomenon regarding disparities within specialties utilizing the Canadian R-1 match data. Second, some data points were missing or unavailable at the time of analysis. Third, we are unable to determine which applicants switched specialty or took a leave of absence, which may influence overall proportions (although we expect these events to be uncommon). Fourth, we were also unable to determine the reasons why CMGs apply to specific programs or for further subspecialization (ie, for fellowships), which were not included in our analysis.

## Conclusions

Trends in the representation of female CMGs among R-1 entry match applicants varied by specialty, with an overall increase over time seen in most, but not all, specialties. When considering applicant success, female applicants were more likely to be unsuccessful in matching to their first-ranked specialty when applying to a surgical specialty and more likely to be successful when applying to psychiatry and family medicine. The reasons for differences in specialty selection and match success by sex require further research, and strategies must be developed to address these disparities. Furthermore, future research should be performed looking at the intersection of gender (for all gender identities) and other self-identified social constructs (eg, race, ethnicity, ability).

## Notes

Supplement. **eTable.** Number of CaRMS Applicants by Specialty, Stratified by Gender
