# Supplementary material for: Trends in Representation of Female Applicants and Matriculants in Canadian Residency Programs Across Specialties, 1995 to 2019
Source: JAMA Netw Open. 2020 Nov 24;3(11):e2027938. doi: 10.1001/jamanetworkopen.2020.27938 (PMC7686870; doi:10.1001/jamanetworkopen.2020.27938)
Supplement: Supplement. — eTable. Number of CaRMS Applicants by Specialty, Stratified by Gender [file jamanetwopen-e2027938-s001.pdf]

Supplemental Online Content

Lorello GR, Silver JK, Moineau G, McCarthy K, Flexman AM. Trends in representation of female applicants and matriculants in Canadian residency programs across specialties, 1995 to 2019. *JAMA Netw Open*. 2020;3(11):e2027938. doi:10.1001/jamanetworkopen.2020.27938

eTable. Number of CaRMS Applicants by Specialty, Stratified by Gender

This supplemental material has been provided by the authors to give readers additional information about their work.

© 2020 Lorello GR et al. *JAMA Network Open.*

eTable. Number of CARMS applicants by specialty, stratified by gender.

Discipline	Total Applicants (n)	Female (n)	Male (n)	Female (%)
**Anesthesiology**	**2,463**	**1,005**	**1,458**	**40.8**
**Emergency Medicine**	**1,673**	**716**	**957**	**42.8**
**Family Medicine Grouping**	**16,473**	**10,333**	**10,582**	**62.7**
Family Medicine	16,224	10,171	6,053	62.7
Community and Public Health	249	162	87	65.1
**Internal Medicine**	**7,905**	**3,942**	**3,963**	**49.9**
Dermatology	783	521	262	66.5
Internal Medicine	7,117	3,420	3,697	48.1
Occupational Medicine	5	1	4	20.0
**Neurology**	**907**	**465**	**442**	**51.3**
**Obstetrics and Gynecology**	**2,090**	**1,776**	**314**	**85.0**
**Pathology & Laboratory Medicine**	**683**	**317**	**366**	**46.4**
Anatomical Pathology	280	125	155	44.6
General Pathology	18	9	9	50
Hematological Pathology	20	12	8	60.0
Laboratory Medicine	234	106	128	45.3
Medical Biochemistry	7	3	4	42.9
Medical Microbiology	119	60	59	50.4
Neuropathology	5	2	3	40.0
**Physical Medicine and Rehabilitation**	**416**	**216**	**200**	**51.9**
**Pediatrics & Medical Genetics**	**3,198**	**2,427**	**771**	**75.9**
Medical Genetics	81	59	22	72.8
Pediatrics	3,117	2,368	749	76.0
**Psychiatry**	**2,810**	**1,663**	**1,147**	**59.2**
**Radiation Oncology**	**394**	**178**	**216**	**45.2**
**Radiology**	**2,055**	**658**	**1,397**	**32.0**
Diagnostic Radiology	1,961	636	1,325	32.4
Nuclear Medicine	94	22	72	23.4
**Surgery**	**7,357**	**2,711**	**4,646**	**36.8**
Cardiac Surgery	206	59	147	28.6
General Surgery	1,909	901	1,008	47.2
Neurosurgery	394	90	304	22.8
Ophthalmology	1,026	382	644	37.2
Orthopedic Surgery	1,303	324	979	24.9
Otolaryngology	742	303	439	40.8
Plastic Surgery	911	423	488	46.4
Thoracic Surgery	5	0	5	0
Urology	773	195	578	25.2
Vascular Surgery	88	34	54	38.6
**Overall**	**48,424**	**26,407**	**22,017**	**54.5**

© 2020 Lorello GR et al. *JAMA Network Open.*
